# Dynamic Grammar Pruning for Program Size Reduction in Symbolic Regression

**DOI:** 10.1007/s42979-023-01840-y

**Published:** 2023-05-17

**Authors:** Muhammad Sarmad Ali, Meghana Kshirsagar, Enrique Naredo, Conor Ryan

**Affiliations:** Department of Computer Science and Information Systems, University of Limerick, Castletroy, Limerick, V94 T9PX Ireland

**Keywords:** Grammatical evolution, Grammar pruning, Production ranking, Effective genome length

## Abstract

Grammar is a key input in grammar-based genetic programming. Grammar design not only influences performance, but also program size. However, grammar design and the choice of productions often require expert input as no automatic approach exists. This research work discusses our approach to automatically reduce a bloated grammar. By utilizing a simple Production Ranking mechanism, we identify productions which are less useful and dynamically prune those to channel evolutionary search towards better (smaller) solutions. Our objective in this work was program size reduction without compromising generalization performance. We tested our approach on 13 standard symbolic regression datasets with Grammatical Evolution. Using a grammar embodying a well-defined function set as a baseline, we compare effective genome length and test performance with our approach. Dynamic grammar pruning achieved significantly better genome lengths for all datasets, while significantly improving generalization performance on three datasets, although it worsened in five datasets. When we utilized linear scaling during the production ranking stages (the first 20 generations) the results dramatically improved. Not only were the programs smaller in all datasets, but generalization scores were also significantly better than the baseline in 6 out of 13 datasets, and comparable in the rest. When the baseline was also linearly scaled as well, the program size was still smaller with the Production Ranking approach, while generalization scores dropped in only three datasets without any significant compromise in the rest.

## Introduction

Grammatical Evolution (GE) is a grammar-based Genetic Programming (GP) approach which has found wide acceptance in the research communities [1, 2]. It is a bio-inspired population-based methodology from the domain of evolutionary computation which heavily relies on the definition of context-free grammars (CFGs). By defining grammar in any language of choice, GE can evolve valid programs of arbitrary length and complexity. This flexibility makes GE a powerful tool in genetic programming (GP) and it has gained a wide-scale appeal.

Grammar is a key input to grammatical evolution and it has been known that the performance of GE is significantly influenced by the design and structure of the grammar [3]. However, there is little guidance in the literature when it comes to defining grammar. This task is generally performed by the users of GE, solutions developers or domain experts, each of whom hand-craft the grammar, often using the same grammar from similar problems. The choice of terminals and non-terminals, and their composition to form production rules, is largely based on expertise. For novice users, there is no tool or framework which can assist them in defining the grammar. A related problem, faced even by experienced users, is the choice of the function set. Functions or operators are represented as productions in the grammar. Choosing an appropriate function set is a key decision in applying GP as it can have a vital impact on the performance of GP [4, 5]. However, there is insufficient guidance in selecting a function set, and no systematic approach exists [6]. To date, it is also largely considered a decision made by domain experts.

Automatic Grammatical Evolution (AutoGE) [7] is a system that can aid users of GE to explore and identify grammar structures to smoothly adapt according to the underlying problem domain. It can aid users in identifying appropriate terminals involved in forming production rules. It is being developed with a rich suite of algorithms that can adapt (prune or extend) user-provided grammar or even generate an appropriate grammar from scratch if certain information about the problem at hand is known. Grammar pruning is one of the key processes used by AutoGE to reduce the size of an *extended* grammar. It has been reported in earlier efforts [7, 8] that pruning can help in significantly reducing program size. Although bloat is not considered as much of a problem in GE as in GP, there are several benefits to size reduction, including efficient evaluation and explainability

This work reports a new approach to grammar pruning, *Dynamic Grammar Pruning*, and applies it to real-world symbolic regression problems. For a given grammar structure and a larger function set, it reduces the grammar by pruning less useful productions. It helps evolve individuals of shorter lengths, thereby optimizing memory usage [9]. We discuss the dynamic pruning algorithm in “Methodology” section. The contents of the remaining sections are as follows: “Background” and “Related Work” sections briefly outline the theoretical background and the related work; “Experimental Setup” section describes our experimental setup; “Results and Discussion” and “Further Analysis” sections present the results and the analysis; and finally, “Conclusion” section concludes our discussion.

## Background

### Grammatical Evolution

Grammatical Evolution (GE) is a variant of Genetic Programming (GP) in which the space of possible solutions is specified through a formal grammar. Although different types of grammar have been used [10, 11] in GE, the most commonly used is Context Free Grammar (CFG), generally written in Backus-Naur Form (BNF). GE facilitates a modular design, which means that any search engine can be used. However, typically a variable-length Genetic Algorithm (GA) is employed to evolve a population of binary strings.

**Fig. 1:**
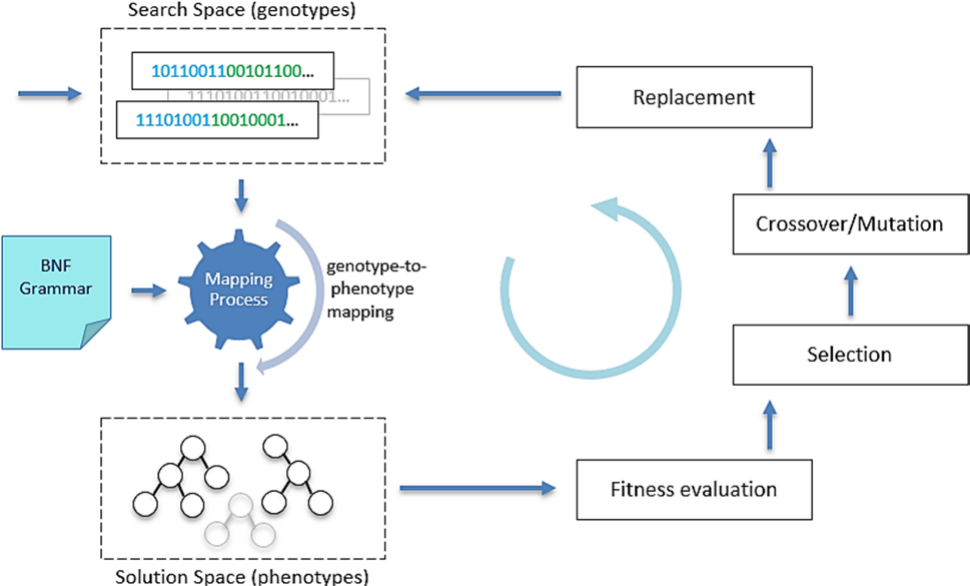
Schematic of evolutionary process in GE

In GE, each individual has a dual representation, a genotype and a phenotype. When the underlying search engine is a genetic algorithm, the genotype is a sequence of codons (usually a group of 8-bit substrings), while the phenotype expresses an individual’s representation in the solution space. Mapping, a key process in GE, maps a given genotype to its phenotype. By consuming a codon, it selects a production from the available set of alternative productions in a rule through mod operations and builds the derivation tree [12]. Although there are other mapping schemes [13], the conventional scheme follows the left-most derivation. An important measure in the mapping process is the *effective genome length*, which is equal to the number of codons consumed to generate a fully mapped individual (which does not contain any non-terminals in its phenotype). The actual genome length is the total number of codons in the genome, some of which may remain unused (Fig. 1).

### Grammar Design

Since GE exploits the expressive power of grammars, it can be applied to a multitude of problem domains, for instance in Symbolic Regression (SR), where the purpose is to search the space of mathematical expressions to find a model that best fits a given dataset [14]. To construct valid and useful mathematical expressions in GE, the grammar needs to be well-designed.

A grammar is formally defined as the tuple (T, N, P, S) where T is a set of terminal symbols, N is a set of non-terminal symbols, P is a set of production rules, and S is the start symbol. While the set of terminals outlines the building blocks of a solution, the choice of non-terminals and deciding how exactly to organize those into a set of rules and productions is a design task. By designing an ‘appropriate’ grammar, one specifies the syntactic space of possible solutions (it is worth noting that there are an infinite number of possible grammars which specify the same syntax). Although grammar design is an important consideration, most research provides little to no justification for design decisions related to choosing (non)terminals and forming production rules.

It is important to note how operators and functions are represented as productions in the grammar. Besides embodying arithmetic operators, a number of common mathematical functions are represented as alternative recursive productions.

### Production Ranking

**Fig. 2:**
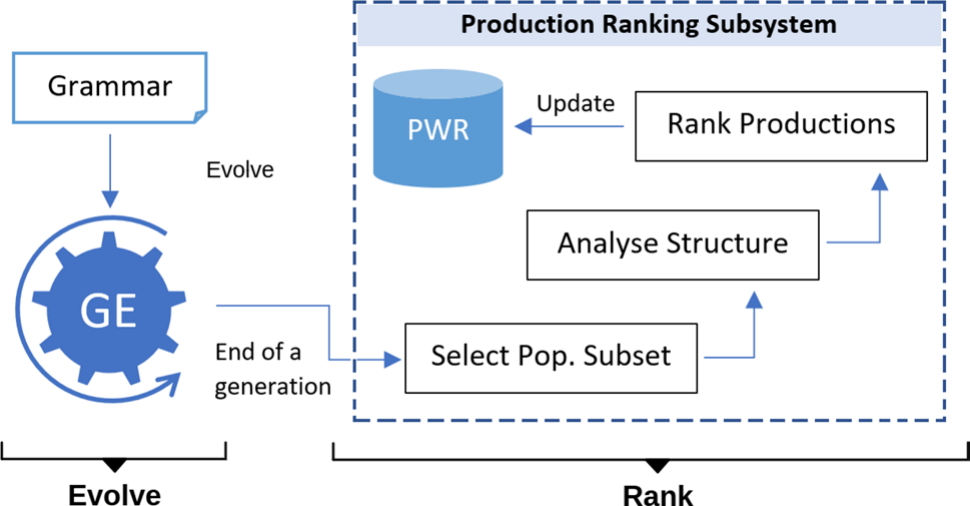
Production ranking process

Production ranking [7] is a process in which grammar productions are assigned numeric scores or ranks based on how frequently they are used to construct individuals in the population. It is based on the hypothesis that the structural composition of evolved solutions carries information that can be useful in identifying useful building blocks for a grammar. As evolution proceeds, the evolved solutions contain an increasing number of useful building blocks (grammar productions in the case of GE) [14]. Fitter individuals survive, and the productions which more frequently shape their structures are those that are considered to be worth being part of the grammar. Such productions are assigned a high rank. Less useful productions which harm the fitness of the population individuals generally do not enjoy high usage frequency during mapping. Figure 2 shows the schematic of the production ranking process. The ‘Evolve’ step encapsulates the regular GE evolutionary cycle, while ‘Rank’ proceeds at the end of a generation. During ranking, a subset of the population (usually a top portion, see Table 2) is selected for structural analysis. Each individual is analysed to identify the list of productions used in its derivation, which we call *production-list*. Ranks of the grammar productions are then computed based on their occurrence frequency and the individual’s fitness. Finally, the ranks are accumulated and stored in a data structure that we call *Production Worth Record* (PWR). What follows is a more formal account of the ranking process.

Let *P* be the set of all productions in the grammar G. $P_i \subset P$ is the subset of productions in the production list of the $i$th individual. Let *n* and *k* be the number of productions in *P* and $P_i$ respectively, then $k < n$ for practically all individuals. The ranking score assigned to the $j$th production in the production list is given by:

1 $$\begin{aligned}{} & {} (n\!f\!r)_i^j = \left( \begin{array}{c} \frac{\phi _i^j}{l_i} \end{array} \right) \end{aligned}$$

2 $$\begin{aligned}{} & {} (f\!pr)_i^j = (n\!f\!r)_i^j \times \rho _i \end{aligned}$$

where $\phi _i^j$ is the frequency of $j$th production, $l_i$ is the effective codon length, and $\rho _i$ is the fitness of $i$th individual. Equation (1) defines the *normalized frequency rank* (*nfr*) of a production, while Eq. (2) computes the *fitness-proportionate rank* (*fpr*). As a consequence of the above two definitions, the following two properties hold for an $i$th individual:

$$\begin{aligned} \sum _{j=1}^k{(n\!f\!r)_i^j}=1,\, \text {and} \quad \sum _{j=1}^k{(f\!pr)_i^j}=\rho _i \end{aligned}$$

Once individual ranking scores have been computed, we accumulate the scores of all *u* individuals in the population to compute *generation worth* (*gw*) of $j$th production in the production-list for $m$th generation, and then across all $g$ generations to compute the overall *run worth* (*rw*).

3 $$\begin{aligned}{} & {} (gw)_m^j = \sum _{i=1}^u (f\!pr)_i^j \end{aligned}$$

4 $$\begin{aligned}{} & {} (rw)^j = \sum _{m=1}^g (gw)_m^j \end{aligned}$$

Due to simple normalization and aggregation operations, the computation of ranking scores is a trivial and thus efficient process. A discussion on the ranking cost and how it can be further minimized is presented in “Cost of Ranking” section.

### Grammar Pruning

According to Occam’s razor, “no more things should be presumed to exist than are absolutely necessary.” Grammar pruning follows this principle in an attempt to limit the complexity of the models and favour simpler ones to take part in the evolution. Grammar is a key solution space model, so the idea is to remove unnecessary or less worthy productions (or functions) from the grammar to *tune* the grammar design. Production ranking is the key enabler for grammar pruning by providing *ranking profile* of the productions, an estimate of productions utility.

**Fig. 3:**
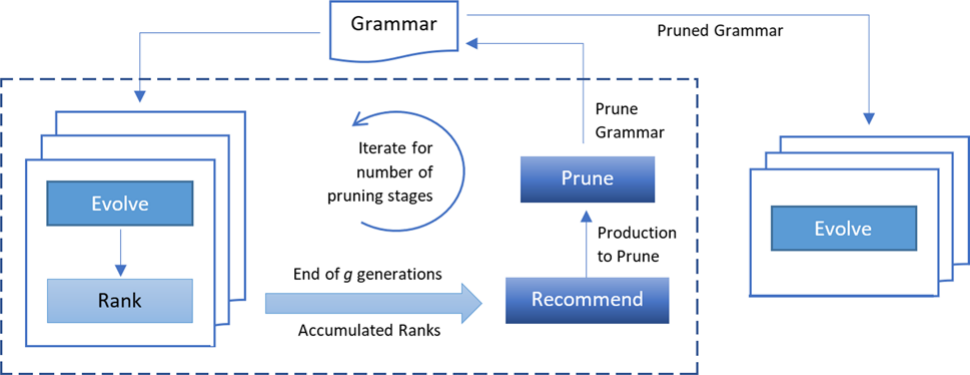
Grammar Pruning process

There can be a number of possible approaches to grammar pruning. The one presented in [7, 8] is a staged approach, depicted in Fig. 3. ‘Evolve’ represents regular GE runs over a certain number of generations. The dotted rectangular region represents the pruning cycle, which continues over the number of stages. Each stage evolves the population over a small number of *g* generations. Production ranking (represented as ‘Rank’) takes place at the end of each generation, as depicted in Fig. 2. At the end of each stage, a recommender system analyses production ranks accumulated over *g* generations and recommends the least ranked production(s) to be pruned. The grammar is then updated by pruning (removing) the identified production and is fed as input to the next stage. The number of stages is kept small (usually 2–4), consuming only a fraction of the budget (20%, for instance). At the end of the pruning process, the final pruned grammar is used for regular GE runs (the ‘Evolve’ cycle in the right of Fig. 3), consuming the rest of the budget (80% for instance).

## Related Work

There are several strands of research and applications relevant to our work. Below we discuss some of the main research directions and present a brief account of some existing literature.

### GE for Symbolic Regression

Symbolic regression searches the space of mathematical expressions to find a model that best fits the given dataset. GE has been extensively used to evolve expressions by randomly combining building blocks (mathematical operators, functions, constants, and variables) represented in the grammar. A good overview can be found in the text of Ryan et al. [2]. Although several works considered standard GE, there are notable efforts for using enhanced GE methods for symbolic regression, for example, Structured GE [15], Geometric Semantic GE [16], $\pi$-GE [17], Hierarchical and Weighted Hierarchical GE [18, 19]. In addition to using simple CFGs, there have been efforts to exploit varying grammar formalisms, for example, GE with stochastic CFG [20], attribute grammars [11], tree-adjoining grammars [21], and Christiansen grammars [10].

### Function Set Selection

It is important to select an appropriate function set in order to achieve good performance in GE/GP. Not many works appeared that specifically address the problem of function set selection. Wang and Soule [4] experimented with various function sets and highlighted that function groups exist and functions in the same group have the same effect on performance. Nguyen et al. [5] examined characteristics of the fitness landscapes generated by various function sets and the performance of GP. Their results indicated that the smoothness fitness landscape generated by a function set measured through autocorrelation could be used as an indicator to select an appropriate function set for a problem. Recently, Nicolau and Agapitos [6] studied the effect of various groups of function sets on the generalization performance of GP and GE. With a detailed review and experimentation over a large set of symbolic regression problems, they concluded that protected functions should be avoided. They also indicated that a category of sets, termed ‘Full Sets’ (the function sets comprising nearly all considered function primitives), performed consistently well in training across all problems. Our earlier studies [7, 8], where we used a larger generic function set at the start of the evolutionary process, support this finding.

### Encapsulation

In a normal setup of canonical GE, grammar is a static artefact which never changes during the execution. However, in this work, we modify the grammar by removing productions from the grammar, which we term as *pruning*. Several strands of research modify the grammar dynamically during the evolutionary process. Instead of striving to choose an optimal set in advance, the idea of automatically defined functions is about identifying, encapsulating, and reusing useful functionality discovered during the evolution [22]. O'Neill and Ryan [23] used a grammar-based approach to automatically define a new function for the Santa Fe trail problem. Harper and Blair [24] introduced a meta-grammar into grammatical evolution, allowing the grammar to dynamically define functions without needing special-purpose operators or constraints. More recently, [25] utilized covariance between traits to identify useful modules added to the grammar.

### Probabilistic GE

In our work, we assign a weight called *rank* to a production, which at a stage is used to decide upon its fate: whether or not to stay in the grammar. Although our ranks do not bias the selection of a production during the mapping process, the probabilistic approach to GE does. It uses probabilistic grammar, also known as stochastic context-free grammar (SCFG), to assign selection probabilities to each production. Although much research explores probabilistic grammars in connection to GP and Estimation of Distribution Algorithms (EDA), there haven’t been many attempts to utilize SCFG in GE with genetic operations, except the recent work from [20].

## Methodology

Our approach is based on production ranking and grammar pruning. Productions with the lowest mean rank across generations are candidates to be pruned. A production with a very low rank is expected to be: (1) least present in the phenotypes^1^. Its presence may dissipate with evolution. (2) present in the low fitness individuals. The probability of such a production being present in high-fitness individuals is considerably low.

For grammar pruning, we devise a different approach. The approach discussed in “Grammar Pruning” section is *static* in the sense that the grammar is pruned a posteriori, i.e. when the evolutionary cycle stops after a small number of generations, or a *stage*. The next stage is a complete restart where the population is reinitialized from scratch to trial regular GE for solution approximation. The only input from the previous stage is the pruned grammar. Although the overall ranking and pruning budget is kept small (usually 20 generations), one of the major drawbacks of this approach is that we lose solutions that evolved over a number of generations which could potentially become good solutions. Secondly, once the pruning stages are over, the regular GE run with pruned grammar evolves solutions for, say 20%, less number of generations. There can be several possible approaches to rectify this. The one we propose in this work is called *dynamic grammar pruning*, or *dynamic pruning* for short.

**Fig. 4:**
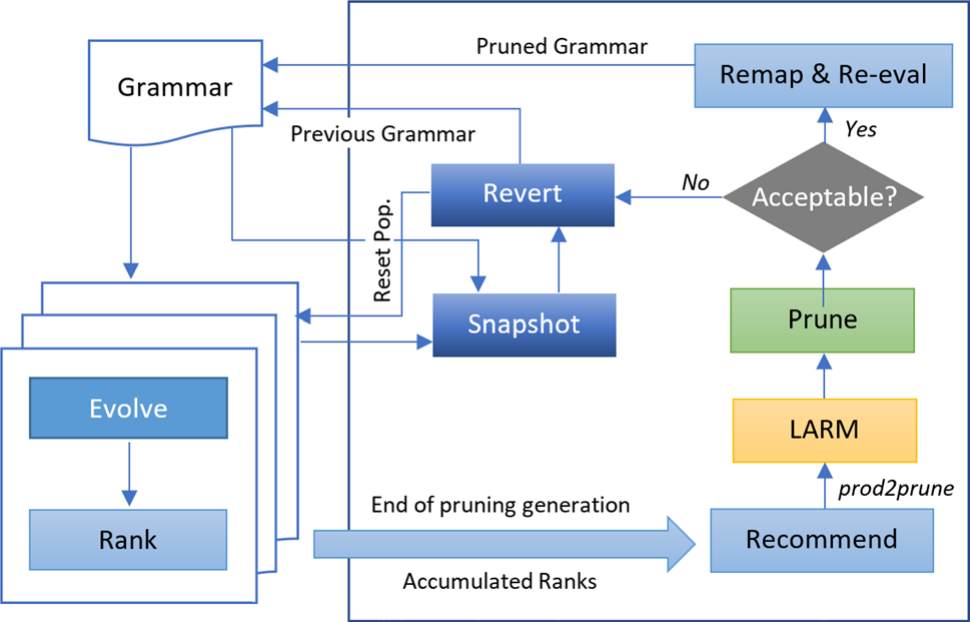
Schematic of dynamic grammar pruning

This section presents our approach and discusses the dynamic production rule pruning (DynPRP) process depicted in Fig. 4.

### Dynamic Pruning

The core idea in dynamic pruning is to prune the grammar during the evolutionary cycle. A production is pruned from the grammar at the end of a generation, and subsequent generations evolve solutions with no knowledge of that production. Let us consider the scenario where, at the end of a generation, we are about to prune a production (hereafter referred to as *prod2prune*) from the grammar. Pruning that production will invalidate the state of the population, which means that for most of the genomes (if not for all), the genotype-to-phenotype mapping will no longer be valid. There are two types of individuals in the population:

- Those which utilized *prod2prune* in the derivation of their phenotype. The mapping will no longer be valid after pruning since the production will not be part of the grammar. A remapping and re-evaluation will be necessary before proceeding with the next generation. However, the new mapping is not guaranteed to generate a valid (fully-mapped) individual. We expect such individuals to be few in the population since our choice of *prod2prune* is based on the lowest production rank;
- Individuals which do not contain *prod2prune*. Their genotype-to-phenotype mapping will also become invalid due to re-indexing the grammar productions. Those individuals are expected to be higher in number and better in terms of fitness scores. We would like to retain those individuals in the population such that a remapping after pruning will not change their phenotype. Consequently, a mechanism is needed to enable this retention.

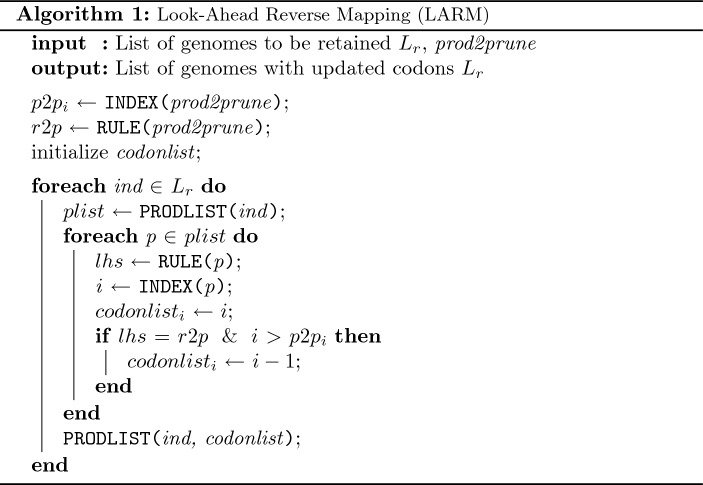


If *prod2prune* is pruned from the grammar, most individuals will not retain their phenotypes in the remapping process, even those which did not contain *prod2prune*. It will cause too much disruption and severely degrade population fitness in most cases. To preserve phenotypes after pruning and remapping, we perform an operation called Look-ahead Reverse Mapping (LARM) which instruments the list of codons so that the codons will correctly remap to the same phenotype. LARM is one of the key functions in DynPRP. The algorithm is presented in Algorithm 1, which simply takes production indexes and assigns those as codon values to the genomes, accounting for the changes in production indices when *prod2prune* will be out of the grammar.

Before LARM, the recommender system is called (shown as Recommend block in Fig. 4), which accumulates the production rank computed at the end of prior generations and identifies a production with the lowest mean run worth. During the search, it ignores productions pruning which would alter the structure of the grammar. The *Prune* function effectively removes the production from the grammar. *Remap* and *Re-evaluate* functions are typically applied to those individuals which contain *prod2prune*.

### Smarter Pruning

Pruning causes disruption as, when genomes are remapped, their fitness can degrade if they had previously used the pruned production rule. Performing a *hard* pruning, without regard to how much disruption a pruning will cause, can negatively affect learning. We do not want to disrupt convergence to the extent that would deteriorate performance, especially generalization. We, therefore, defined a threshold, *acceptable drop in mean population fitness*, to decide whether or not to commit to a particular pruning event. If the drop in mean population fitness is higher than the threshold, the pruning decision is *Revert*ed. To ensure that we revert to the initial state, we define a *Snapshot* function. This brings back both the population and the grammar to their last state.

In dynamic pruning, evolution starts as normal using the extended grammar (see “Extended Grammar” section). After a certain number of generations, pruning comes into operation and takes place at specified generations, known as *pruning generations*, which are determined by three parameters:

1. ($gen_{b4prn}$), which defines how many generations to evolve before pruning begins;
2. $P_{int}$ is the number of generations in between two successive prunings;
3. Number of pruning stages *S*.

These parameters are used to populate the list of pruning generations $P_{gen}$. With the parameter values shown in Table 2, the list becomes [5, 10, 15, 20]. The DynPRP algorithm is therefore invoked whenever the current generation $gen_{curr}$ is in the list of pruning generations, i.e. on 5th, 10th, 15th, and 20th generation, in every run.

## Experimental Setup

We conducted a large set of experiments to study the effectiveness of our grammar pruning approach to reduce program size. The details of the experimental settings, the dataset used, and the grammars embodying various function sets are presented in this section.

### Datasets

The real-world symbolic regression benchmark problems considered in this work are listed in Table 1. All of the 13 problems have been widely exercised in numerous genetic programming-based symbolic regression efforts; some of the recent ones include [6, 26–28].

The collection of datasets is diverse, including problems having 5 to 241 input features, with sample size varying from 60 to nearly 5000. The high variability in terms of the number of features and the number of samples among the chosen datasets was a deliberate attempt to investigate how much the outcome of our approach could be generalized across a variety of problems. Each dataset is referred to with a short name (in a distinct font), which will be used in the rest of the paper. No preprocessing was done on the datasets (except for two, as noted below) while we utilised the raw values without normalization. A few more details are worth mentioning here:

**Table 1:** Datasets used in experimentation

Dataset	Short name	Features	Instances
Airfoil self-noise	airfoil	5	1503
Energy efficiency—heating	heating	8	768
Energy efficiency—cooling	cooling	8	768
Concrete strength	concrete	8	1030
Diabetes progression	diabetes	10	442
Wine quality—red wine	redwine	11	1599
Wine quality—white wine	whitewine	11	4898
Boston housing	housing	13	506
Air pollution	pollution	15	60
Tower	tower	25	4999
Dow chemical	dowchem	57	1066
Communities and crime	ccrime	127	1994
Human oral bioavailability	bioava	241	359

- The following datasets have been sourced from UCI Machine Learning repository^2^: airfoil, heating, cooling, concrete, redwine, whitewine, ccrime;
- These datasets have been acquired from CMU StatLib Dataset Archive^3^: housing, pollution;
- The diabetes dataset, originally used in [29], was obtained from that paper’s co-author website^4^;
- The tower dataset was collected from PonyGE2 github repository^5^ where it is provided as one of the example datasets. We used the version which contained all 25 input variables without any preprocessing;
- The dowchem dataset was acquired from GPBenchmarks.org^6^ website.;
- The ccrime dataset originally contained 127 input features including the target feature. Five features are non-predictive, while 22 features contain missing values in more than 80% of the samples. Removing non-predictive and features with missing values, the dataset contained 100 input features;
- The bioava dataset was obtained from the authors of [30]. It originally contains 241 features. Dick et al. [31] conducted a detailed analysis of this dataset. There are 47 features which contain the value 0 (zero) across all 359 samples. Since those features provide no useful information for learning, we removed those in preprocessing, leaving the dataset with 194 features.

### Parameters

All notable hyperparameters for the evolutionary runs are presented in 2. In all experiments, with each grammar type, we performed repeated 10-fold cross-validation with a repeat factor of 3. For repetition, we used a different seed to ensure a different training-test data split was generated each time. The repetition ensured that we further minimized the chances of bias and overfitting [32]. To generate data folds, we used the KFold() function in *scikit-learn* Python library.^7^ Each experiment was run 50 times to ensure that any conclusions drawn from the results were statistically sound [33].

**Table 2:** Experimental settings

Method	Hyperparameter	Value
GE	Population size	500
	Number of generations ($g$)	100 (25 with linear scaling)
	Number of runs	30
	Search engine	Steady-state GA
	Cross-validation	10-fold
	Crossover type	Effective crossover [34]
	Crossover probability	0.9
	Mutation probability	0.01
	Selection type	Tournament
	Initialization method	Sensible initialization [35]
Pruning	Pop. subset for ranking (*K*)	Top 20%
	Number of stages	4
	Productions pruned per stage	1
	Generations before pruning	5
	Pruning interval	5
	Acceptable fitness drop	10%

The search for good values for the dynamic pruning hyperparameters was carried out empirically by performing a limited grid search. A number of experiments were conducted on a few datasets by varying the parameters in certain ranges, for instance, the number of generations before pruning in the set [5, 10, 15, 20], interval between two successive pruning stages in the set [5, 10], and the acceptable performance drop in the population in the set [5%, 10%]. The parameter values least affecting the generalization performance were chosen, and are shown in Table 2.

We ran all experiments on the libGE system,^8^ which is an efficient C/C++ implementation of canonical GE and which provides capabilities to effectively examine grammar productions. The effective codon length was used as a measure to assess program size. To measure the performance, both in training and test, Root Mean Squared Error (RMSE) was used, which is a common fitness function in symbolic regression. It is defined as:

$$\begin{aligned} \text {RMSE} = \sqrt{\frac{1}{n}\sum _{i=1}^{n}(\hat{y}_i - y_i)^2} \end{aligned}$$

where $n$ is the number of data points, $y_i$ is the target value, and $\hat{y}_i$ is the predicted value. The goal is to minimize RMSE on test data, in addition to reducing program size using grammar pruning. During evolution, to reduce the possibility of introducing biases in selection, we curb candidate solutions producing extravagantly large errors by defining an error ceiling threshold (RMSE $> 10,000$). Any candidate for which fitness evaluates as inf (infinite value), nan (not a number), or beyond the threshold, we assigned minimum fitness.

### Grammars and Function Sets

Since this work is an extension of the work presented in [8] where the use of *mixed-arity* grammar structure was found to be producing minimum effective genome lengths, we utilize the same structure in this work. In terms of the function set embodied in the grammar, we defined *extended* version of the grammar and a *baseline* grammar to compare it with.

#### Baseline Grammar

Nicolau and Agapitos [6] defined a large collection of the function set variants and found that the function sets which they termed ‘Balanced Sets’^9^ were the best performers when it comes to improving generalization performance in GP as well as GE. Building on their results, we defined the following baseline grammar in this work:
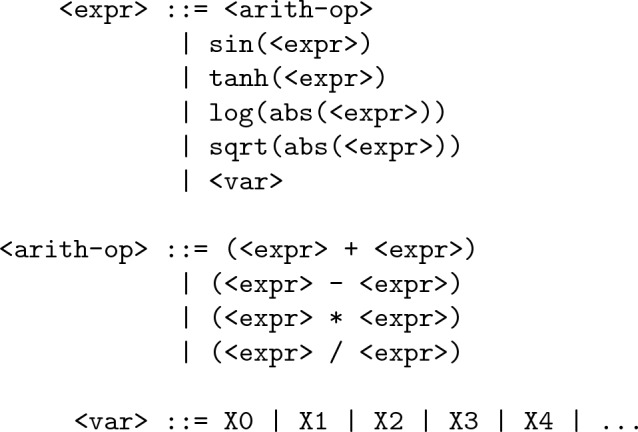


In the above grammar, the <expr> rule is the start rule. The <var> rule carries as many alternative terminal productions as there is the number of input features in a given dataset. The <arith-op> rule models arithmetic operators. Note that in <arith-op>, we model the division operator as an unprotected division (UPD). Although in GP literature, the use of protected division is common, it has been found to be problematic due to the presence of asymptote $\lim _{x_2 \rightarrow 0} x_1/x_2=\infty$. As a remedy, Keijzer [36] proposed using interval arithmetic to probe the regions around training points for discontinuities. Others suggested using un-protected division [36]. Ni et al. [37] proposed a new operator called Analytic Quotient (AQ), which produced better test performance over a variety of synthetic benchmark functions. It is defined as:

$$\begin{aligned} AQ(a,b)=\frac{a}{\sqrt{1+b^2}} \end{aligned}$$

Nicolau and Agapitos [6] also report better generalization performance when using AQ operator, though a large collection of their benchmarks were also synthetic functions. However, it has been noted that using the AQ operator can lead to generating larger trees in GP [37], which is contrary to our objectives. Therefore, to study the effect of using AQ and UPD in the grammar on generalization performance and the genome length in GE, we used two versions of the baseline grammar. The first version, represented as grB$_{dv}$, is shown above. The second version, represented as grB$_{aq}$ uses the same grammar except that the production (<expr> / <expr>) in the rule <arith-op> is replaced with (<expr> / sqrt(1 + pow(<expr>, 2))) which models AQ operator. We conducted experiments with both versions to identify which grammar to choose as a suitable baseline, which will act as a performance baseline for all comparisons.

#### Extended Grammar

In our earlier work [7, 8], we defined an *extended* function set which contained all mathematical functions commonly used in symbolic regression. It included arithmetic operators, trigonometric functions, exponential, and power functions. Besides commonly used functions and operators, we also included some divergent exponential functions such as $e^{-x}$, $\tan (x)$, $\sinh (x)$, and $\cosh (x)$ which grow exponentially and are usually avoided. However, we kept those in our function set in order to validate if our approach of production ranking and grammar pruning was able to remove such functions from the grammar when not useful. Our successes with grammar pruning lead us to present a real challenge to the approach. Therefore, we do not include these divergent functions in the current work. Rather, a restricted set is considered as an extended function set, which includes the following functions: $x^2$, $x^3$, $\sin (x)$, $\cos (x)$, $\tanh (x)$, $e^x$, $\log (|x|)$, $\sqrt{|x|}$. The choice of these functions is based on the survey of GP literature conducted by Nicolau and Agapitos [6]. All of these functions have been used in reputed journal publications in GP. The corresponding grammar which contains productions embodying these functions is called *extended grammar* abbreviated as grE in this work. The grammar is similar to the baseline grammar defined in  “Baseline Grammar” section, except that the <expr> rule is written as below:
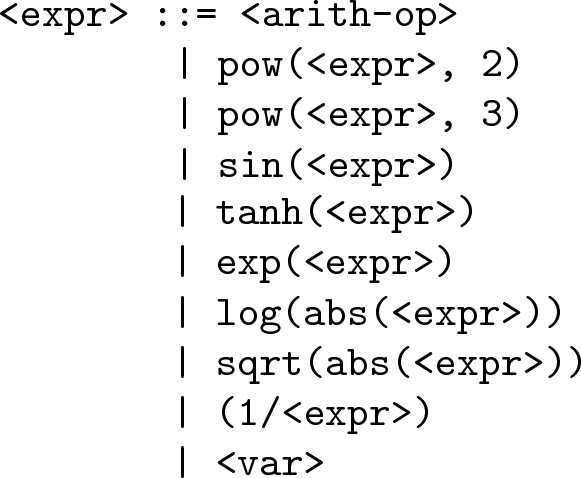


Note that we did not use the protected power function ($x^y$). Rather, we defined two simple unprotected power functions, square ($x^2$) and cube ($x^3$). The inverse (1/*x*) and exponential function ($e^x$) are also unprotected, while we use protected versions of *log* and *sqrt* functions.

## Results and Discussion

We conducted two sets of experiments over 13 datasets: the first set ran without linear scaling, while in the second, we enabled linear scaling for the first 20 generations. In total, we conducted 100+ experiments, where each experiment comprised 50 independent runs for a given dataset and the grammar type. Table 3 presents results from the first set of experiments, while Table 4 presents a summarized view of the outcome from the second set. The presentation details, the rationale of our design decisions, and the outcome are discussed in this section.

### Presentation and Statistical Comparison

Both Tables 3 and 4 present effective size and test performance results for each dataset with three different grammars used as input to GE: baseline grammar with unprotected division operator (grB$_{dv}$), baseline grammar with analytic quotient operator (grB$_{aq}$), and extended grammar (grE). We applied grammar pruning on the extended grammar (grE) and the pruning outcome is represented as grP in the table headers. For each row of the results, the number outside the parenthesis corresponds to the median score among 50 best-of-run solutions. In contrast, the number inside the parenthesis is the median absolute deviation (MAD). Although reporting mean results has been common in the literature, more studies now rely on median results since the median is a more robust measure of centrality in the presence of outliers [38, 39].

We performed tests of statistical significance to compare the output using baseline, extended and pruned grammar. All comparisons were made with respect to the chosen baseline. We used the Mann–Whitney *U*-test (two-tailed version), which is a non-parametric test, with the null hypothesis that, for the randomly drawn samples *X* and *Y* from two independent populations, the probability of *X* being greater than *Y* is equal to the probability of *Y* being greater than *X*. Since the assumption of normality and dependence, as required by parametric tests, does not hold in general in experimental results of evolutionary computing approaches, non-parametric tests are the right choice [39]. All results were compared at the 0.05 statistical significance level. When the *p*-value is below 0.05, we conclude, with 95% confidence, that the compared samples are drawn from different distributions.

In Tables 3 and 4, the blue column indicates the results from the chosen baseline. Where the numbers are in bold, it shows that there was a statistically significant difference between the current result and the baseline. When we present numbers in regular font, without any highlight, this is to indicate that the null hypothesis was not rejected in the statistical significance test. When a cell is yellow-highlighted, it indicates that the results are significantly **better** than the baseline, while in the case of a grey-filled cell, the results are statistically **worse**.

Table 5 lists the percentage gain or drop (shown with either no sign or a negative sign, respectively) in median effective size and median test scores compared to the baseline.

### Choice of Baseline Grammar

Our earlier research has revealed the positive impact of grammar pruning on program size reduction [7, 8]. In this work, we wanted to present a harder problem of grammar pruning with a more realistic function set modelled in the extended grammar. Since our objective was genome length reduction without compromising on generalization performance, we decided to choose a baseline grammar which could provide good results on test data.

As evident from Tables 3 and 4, we chose different baseline grammars in both sets of experiments. In the first set of experiments, we chose grB$_{aq}$ as the baseline grammar since we expected it to perform well in the test, learning from the outcome of other research works [6, 37]. At the same time, we expected grB$_{aq}$ to produce larger individuals [37]. To verify this, and to evaluate how using the UPD operator would perform in comparison to the AQ operator, we ran experiments using grB$_{dv}$ with the same settings and the training/test data folds that were used for grB$_{aq}$. From the results in Table 3 it is clear that both grammars performed well in the test with very similar results across all datasets, except for airfoil where grB$_{dv}$ produced significantly better output. When comparing the effective size of the best solutions, the results are comparable on 10 datasets, while grB$_{dv}$ significantly outperformed grB$_{aq}$ on three datasets. Therefore, to raise the bar in our second set of experiments, we chose grB$_{dv}$ as the baseline.

**Table 3:**
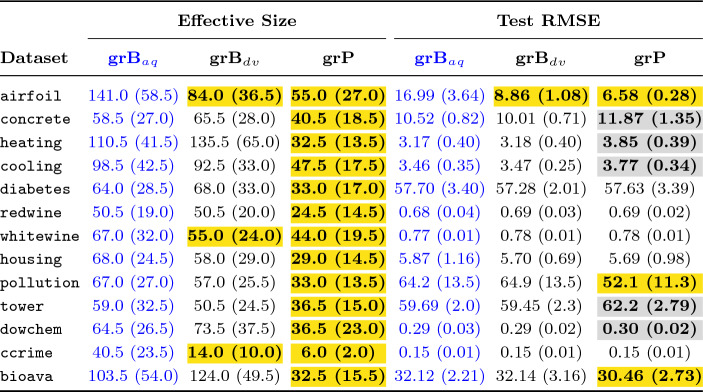
Effective size (genome length) and test RMSE comparisons using baseline, extended, and pruned grammars

The numbers in each cell represent the median and median absolute deviation (in parenthesis)

### Outcome of Grammar Pruning

We applied dynamic grammar pruning in both sets of experiments on all datasets. In the first set of experiments, when comparing the results of dynamic pruning (column grP) with the baseline (column grB$_{aq}$) in Table 3, it is clear that pruning significantly reduced effective genome length in all datasets. The mean percentage improvement in size across 13 datasets is 51.87% (see Table 5), which is fairly high. This greatly fulfils our first goal, the reduction of program size. However, our goal is two-fold: program size reduction without compromising generalization performance. From the right half of Table 3, it is evident that grP successfully fulfilled the second criteria for more than half of the problems. The test RMSE in 8 out of 13 datasets is either significantly better or comparable. In fact, there is a high gain in generalization performance for airfoil and good improvement in the case of pollution dataset.

However, there is a significant drop in test scores for five datasets, more than 10% in the case of concrete and heating. This raised a concern and led us to investigate what aspect of our pruning approach resulted in this drop. Our immediate suspicion was the overzealous pruning of genuinely important productions. When we examined the productions pruned at different stages across all runs for heating, the top four frequently pruned productions were sin(<expr>), cos(<expr>), log(abs(<expr>)), and tanh(<expr>), which are generally expected to be useful productions and commonly used in GE for symbolic regression. A similar pattern was exhibited in the case of cooling, tower, and dowchem. It turned out, for these datasets, there was not enough variation in the population, when evolving for just five generations, to better exploit potentially useful productions. Increasing the number of ranking generations before pruning did not help maintain generalization performance since it reduced the number of post-pruning generations.

We encountered a similar problem in our earlier work [40] when we strived to improve the ranking profile to identify relevant features. Our experimentation revealed that using linear scaling helps to improve the ranking profile and select the ‘right’ terminal production. Linear scaling [36] is a well-known approach in GP. It finds the coefficients (slope and intercept) for the candidate solution, relieving GP of searching for the constants/coefficients. GP can therefore focus on finding “*expression whose shape is most similar to that of the target function*” [36]. We hypothesized that finding the ‘right’ shape would let GP form expressions with the ‘right’ ingredients, and therefore decided to utilize linear scaling in the dynamic pruning approach.

**Table 4:**
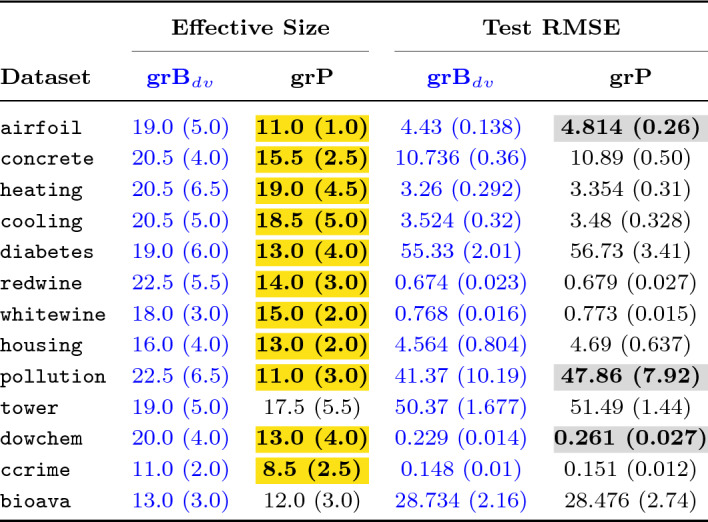
Experimental results with Linear Scaling enabled in first 20 generations for baseline as well as grammar pruning

The numbers in each cell represent the median and median absolute deviation (in parenthesis)

#### LS-enabled Grammar Pruning

At the end of the first set of experiments, we trialled LS-enabled grammar pruning, i.e. LS enabled only during ranking and pruning (first 20 generations). This dramatically improved results. Not only was the effective genome length in all datasets significantly better, test performance significantly improved for six datasets, while it was comparable in the other seven datasets^10^ This was the perfect outcome we strove for. Although we disabled linear scaling once the pruning stages are over, there was, however, one major concern. Linear scaling is known to significantly improve approximation/training performance [36]. Although it does not guarantee improvement in generalization, in the longer run, solutions in most cases either perform better or are comparable on test data [41]. Also, genome lengths of linearly-scaled individuals are relatively smaller. Therefore, LS-enabled pruning had something of an advantage and was somewhat bound to be better in comparison to plain non-LS GE runs with grB$_{aq}$. A fairer level playing comparison would provide LS to the baseline grammar as well.

Another important observation in LS-enabled pruning trials was that the fitness and test performance achieved in the first 20 generations could rarely be surpassed by regular evolution in the later 80 generations.^11^ Therefore, we decided to carry out our second set of experiments for only 25 generations, considering grB$_{dv}$ as baseline grammar (as discussed in “Choice of Baseline Grammar” section). Table 4 presents the results using linear scaling. Once again, the results were promising since grammar pruning improved effective size in all datasets, though improvements in only two cases were insignificant. In 10 out of 13 datasets, pruning only slightly affected test performance. However, in three datasets, the test scores were statistically significantly worse.

**Table 5:** Percentage change in effective genome length and test performance using grammar pruning

	Effective size		Test performance	
Dataset	Without LS	With LS	Without LS	With LS
airfoil	**60.99%**	**42.11%**	**61.22%**	**−7.46%**
concrete	**30.77%**	**24.39%**	**−12.86%**	−1.43%
heating	**70.59%**	**7.32%**	**−21.75%**	−2.88%
cooling	**51.78%**	**9.76%**	**−9.17%**	1.25%
diabetes	**48.44%**	**31.58%**	0.13%	−2.53%
redwine	**51.49%**	**37.78%**	−1.62%	−0.74%
whitewine	**34.33%**	**16.67%**	−1.42%	−0.65%
housing	**57.35%**	**18.75%**	3.05%	−2.76%
pollution	**50.00%**	**51.11%**	**18.90%**	**−15.69%**
tower	**38.14%**	7.89%	**−4.27%**	−2.22%
dowchem	**27.91%**	**35.00%**	**−6.99%**	**−13.97%**
ccrime	**83.95%**	**22.73%**	−2.67%	−2.03%
bioava	**68.60%**	7.69%	**5.17%**	0.90%
Average	51.87%	24.06%	2.13%	−3.86%

Reduction in effective size and the test error is represented with a positive number, while increase with a negative number. Bold indicates statistical significance

## Further Analysis

Apart from the results and discussion above, it is important to present an analysis of which productions were pruned and how much computational overheads were incurred in the process. Below, we outline those details.

### Productions Pruned

The grammar pruning strategy utilized in this research is dynamic pruning (detailed in “Methodology” section). Each pruning stage pruned a different production from the grammar, as suggested by the recommender module. The chosen acceptable fitness drop level was set to 10%. Recall that, in dynamic pruning, when pruning a production resulted in more than 10% drop in the mean population fitness, the pruned production was reverted. Therefore, the number of productions pruned in each run of an experiment varied from 0 to 4, out of 9 prunable productions in grE.

**Fig. 5:**
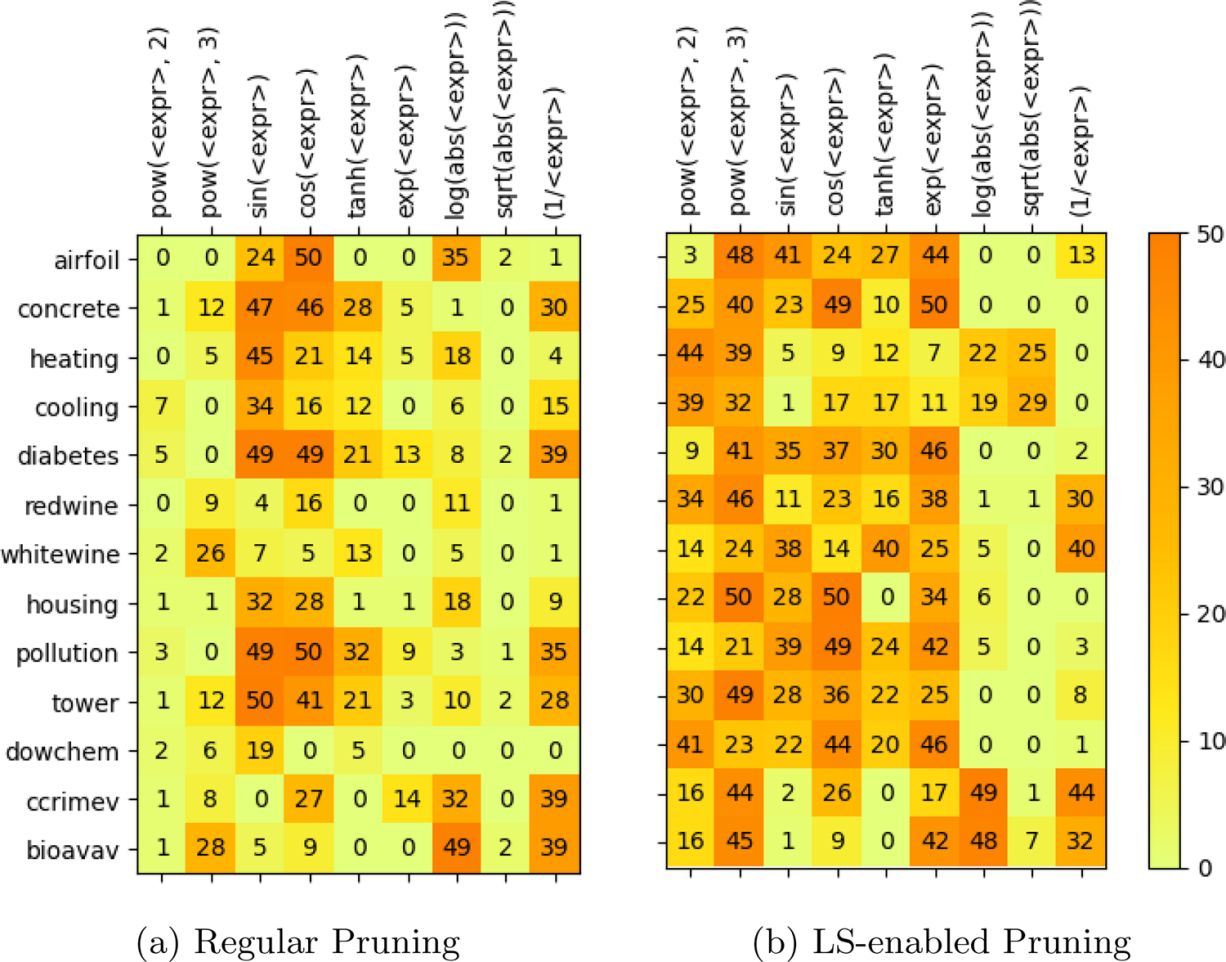
Heatmaps showing the cumulative frequency of productions pruned in four stages over 50 runs across all datasets

We logged a wealth of relevant information for each experimental run with grammar pruning; for instance, which production was pruned at each stage, how it affected the population, and how much time it took, etc. Since it would not be feasible to expose fine-grained details, we present a summarized view of prunings here in the form of a heatmap. Figure 5 provides two heatmaps: (a) against the first set of experiments (without LS), (b) against the second set (with LS). The number in each cell counts how many times a particular production was pruned in all 50 runs (in any of the four stages) for a particular dataset. A few observations are notable here:

- The number of productions pruned in regular pruning is much less than LS-enabled pruning, which pruned a production at almost every stage. The average number of productions pruned per dataset in the case of LS-enabled pruning is 192.77 (out of 200 possible pruning), and 115.9 in the case of regular pruning;
- There is a clear contrast between frequently pruned productions when pruning with and without LS. The top four heavily pruned productions in case of regular pruning are: sin(<expr>), cos(<expr>), (1/<expr>), log(abs(<expr>)). The top four productions pruned in case of LS-enabled pruning are: pow(<expr>, 3), exp(<expr>), cos(<expr>), pow(<expr>, 2).

There are a couple of questions which demand a more detailed examination: Are there any patterns in the choice, order, and rank of pruned productions which negatively affect generalization performance? With the current ranking scheme, how can we improve the pruning suggestion mechanism? How does static pruning compare with dynamic pruning? Studies are being planned to investigate and answer these questions.

### Computational Overhead

Both production ranking and grammar pruning introduce some overhead in computation. These overheads include both time and space complexity costs. Below, we present an analysis of costs in terms of execution times. Memory overhead analysis is one of the future tasks in our research. However, we did put considerable effort into our implementation to reduce spatial complexity and reclaim additional memory allocations once the ranking and pruning iterations were over.

**Fig. 6:**
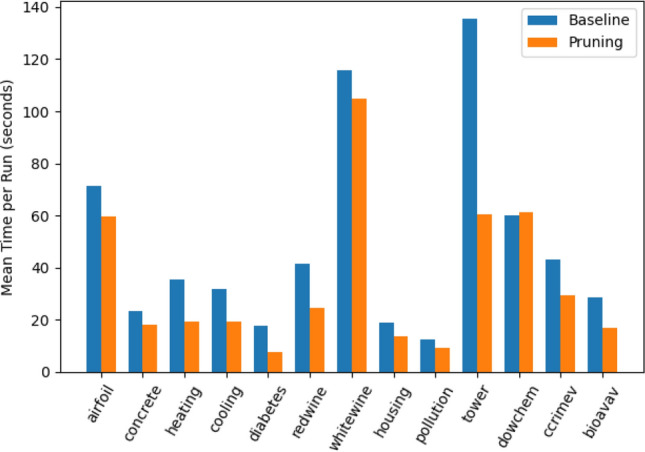
Mean time to execute a single run (100 generations) with the baseline grammar (grB$_{aq}$) and grammar pruning

Before we delve into a more detailed discussion about the cost of ranking, pruning and linear scaling, we present an overall comparison. The bar plot shown in Fig. 6 provides a snapshot of the average run time when using baseline grammar (grB$_{aq}$) and with pruning. Except in the case of dowchem, execution time with grammar pruning is considerably less than when using plain GE with baseline grammar. When the 13 samples of the mean execution times in both cases are compared using a Wilcoxon Signed-Rank test for paired values, the difference is highly significant at a *p*-value of 0.00188. Therefore, we conclude that our approach carries not only negligible overheads but also provides significant gains in execution times.

#### Cost of Ranking

In our system, production ranking is implemented as a plugin feature. Once enabled, ranking starts accumulating production ranks (as detailed in “Production Ranking” section) until a specified number of generations (20 in our experiments). The overhead of production ranking increases the computational cost incurred in analysing the individuals during the evolution. Time expended in ranking depends on two measures: the population size and the size of the individuals. An individual with a larger size (effective codon length) would have more productions utilized in its derivation, which would cause the ranking algorithm to take more time to log their usage frequency and compute fitness-proportionate ranks. Note that we are analysing only the top 20% of the population, which means only one-fifth of the individuals are structurally analysed. To further minimize computational overhead, we make use of certain implementation-specific actions:

**Fig. 7:**
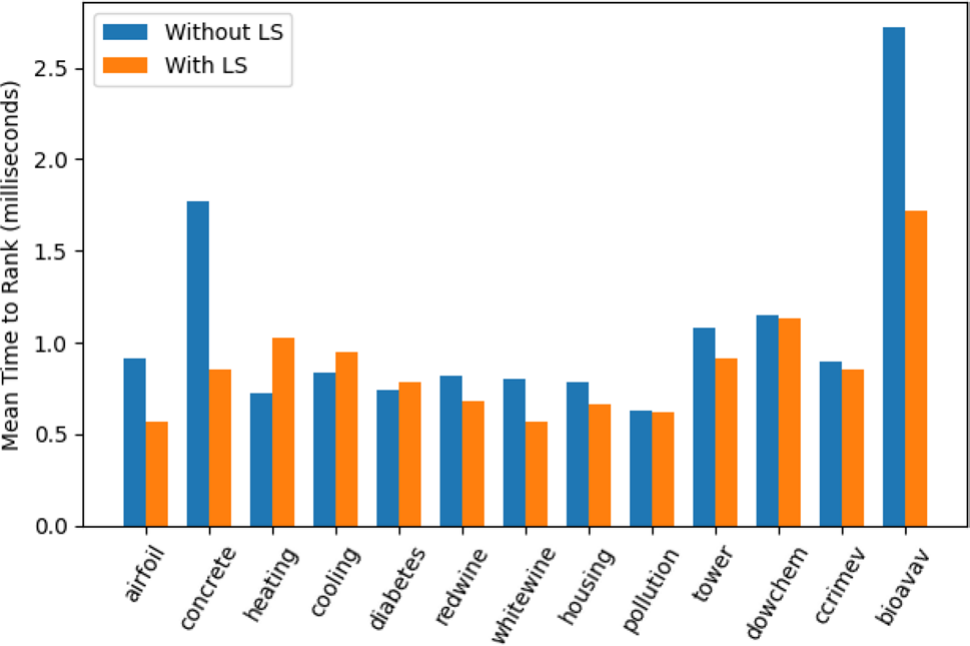
Mean time to compute production ranking scores per generation for all the datasets

- We attach the list of productions to the individual during the mapping process. Although this requires some extra bytes per individual, it helps in avoiding remapping or regenerating derivation trees to identify productions;
- A single data structure (2-D array) keeps track of the data (termed *production worth record*) during ranking computations.

Figure 7 shows the time (in milliseconds) spent in computing production ranking scores per generation averaged over 50 runs, with and without linear scaling, for all datasets.^12^ The mean ranking time per generation (averaged over the first 20 generations) across all datasets is 0.87 ms with linear scaling and 1.06 ms without linear scaling. The cost of ranking without linear scaling is generally slightly higher than with linear scaling, since the effective size is relatively smaller with linear scaling. However, there are a few observations. The with-LS ranking cost in case of heating, cooling, and diabetes is slightly higher. Similarly, the ranking costs in the case of concrete and bioava are relatively higher compared to other datasets. A possible explanation could be the presence of larger individuals in the population subset used for ranking. However, a detail investigation is needed to examine these occurrences.

#### Cost of Pruning

**Fig. 8:**
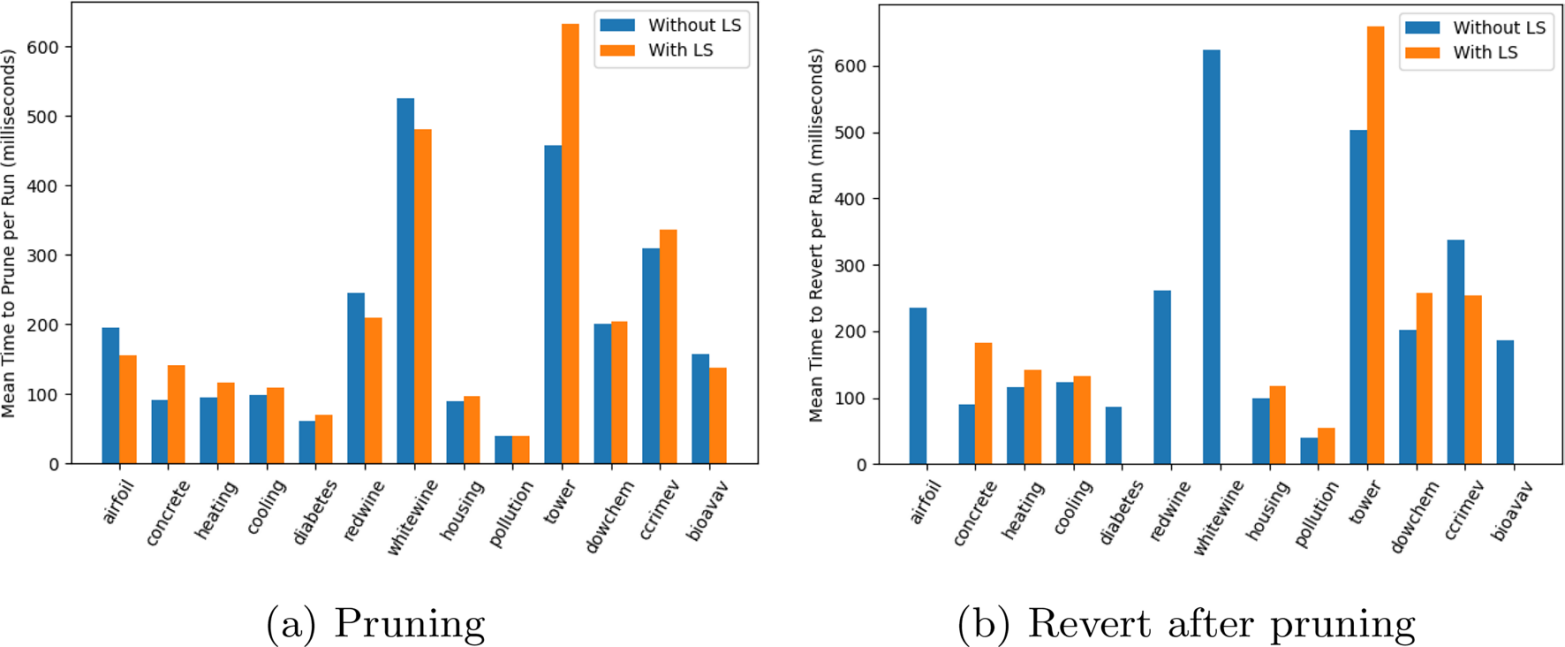
Mean time to prune and revert per run runs across all datasets

Figure 8 shows the average cost of pruning per run per dataset. As detailed in “Methodology” section, dynamic pruning comprises a number of functions: *Recommend*, LARM, *Prune*, *Remap*, *Re-eval*, *Snapshot*, and *Revert*. In general, the most expensive function among these is *Re-eval*, while the least expensive ones are *Prune* and *Snapshot*. From Fig. 8, one can note that the mean time to prune and revert is proportional to the number of data samples in the dataset. Both whitewine and the tower datasets contain nearly 5000 instances, while pollution contains only 60. Below are a few more observations:

- During the initial stages of pruning, there are very few individuals which are affected. In turn, there are fewer *Remap* and *Re-eval* calls. Intuitively, the cost generally increases at later stages;
- In our implementation of LARM, entirely replacing the list of codons was computationally more efficient as compared to finding new codons values and rectifying the relevant sequence;
- The higher the number of Revert calls, the higher the cost of pruning, as there are extra operations to undo the pruning actions. To make it more efficient, we take a *Snapshot* of the population and the grammar, though it is spatially inefficient.
- Revert calls happen for every dataset when pruning without LS. However, with LS, revert does not take place for five datasets. On the other hand, the cost to revert is higher with LS since production pruning frequencies are higher in the case of LS-enabled pruning.

## Conclusion

We proposed a new grammar pruning algorithm, dynamic grammar pruning, as part of the AutoGE tool suite being developed. Using production ranking mechanism to identify less worthy productions, it successively reduces the size of grammar to drive the evolutionary search towards shorter and often better solutions. We tested our approach on a diverse set of real-world regression problems and found that the evolved solutions were significantly smaller in size for all datasets.

While reducing program size, improving or maintaining generalization performance is a challenge. Our results with regular dynamic pruning were promising. Program size was significantly reduced in all datasets, while test performance in 8 out of 13 datasets was either significantly better or comparable, though it worsened in five datasets. We utilized LS only during the production ranking stages (first 20 generations) to generate an improved ranking profile. When comparing LS-enabled dynamic pruning with linearly scaled baseline, the program size outcomes persisted. At the same time, the generalization scores dropped in only three datasets without any significant compromise in the rest of the 10 datasets. These results led us to conclude that dynamic grammar pruning is a promising approach which can not only be applied for program size reduction but primitive set identification as well.

### Future Directions

There are several possible extensions to our work; a few are already in process, while others are being planned. Some of the ideas are mentioned below:

- The current work can be extended with both function and feature selection in a parallel pruning fashion to exercise high-dimensional problems. Although we have used a few relatively high-dimensional regression datasets, we intend to exercise much higher levels of dimensionality (500+ features).
- Besides, classification problems can be exercised with the grammar pruning approach. For high-dimensional classification problems, we can utilise our grammar-based feature selection approach [40]. On the other hand, symbolic classification [42, 43] can be helpful in multi-class classification;
- We want to improve *Recommend* function in dynamic pruning. The current production ranking mechanism is efficient but fairly simple and unintelligent. Utilizing an ML approach to better predict which production to prune will be an interesting study;
- Another improvement to the production ranking scheme can be to employ more opportunistic production ranks to assess marginal contributions of each production in the solution using approaches such as variants of Shapely-Value [44], without adding much computational overhead;
- It would be interesting to explore how DynPRP would perform with other variants of GE or grammar-based GP approaches.
- We also intend to explore other problem domains, for instance program synthesis and Boolean logic.

## Footnotes

1 Note that a fully-mapped individual’s phenotype does not contain any productions; rather the productions are used in the derivation of its phenotype. For example, an individual with the phenotype sin(1 + sqrt(X)) utilizes the production sin(<expr>) in its derivation. However, since the non-terminal part of the production sin( ) appears in the phenotype after derivation, we sometimes take the liberty of phrasing it as ’phenotype contains production’, though not in the literal sense.

2 https://archive.ics.uci.edu/ml/datasets/communities+and+crime.

3 http://lib.stat.cmu.edu/datasets/.

4 https://hastie.su.domains/Papers/LARS/.

5 https://github.com/PonyGE/PonyGE2/tree/master/datasets.

6 http://gpbenchmarks.org/?page_id=30.

7 https://scikit-learn.org/stable/modules/generated/sklearn.model_selection.KFold.html.

8 http://bds.ul.ie/grammatical-evolution/.

9 In [6], Balanced Sets are a category of function sets having different combinations of $\log (|x|)$, $\sqrt{|x|}$, $\sin (x)$, $\tanh (x)$, besides mandatory $+$, −, and $*$. Note that these sets do not include (protected) division. Nor do these include inverse, power and exponent functions. In that work, ‘Balanced Sets with aq’ are the Balanced Sets with the addition of the analytic quotient operator.

10 This outcome was achieved when we compared test RMSE scores with grB$_{aq}$ (shown in Table 3) with the corresponding scores for LS-enabled pruning (grP shown in Table 4).

11 Note that in our experiments with GE, the underlying GA was steady-state, which was unaware of any form of scaling and carried genetic operations as normal. Once evaluated, a linearly-scaled individual’s slope and intercept values were only part of an individual’s metadata.

12 Note that this time does not include the time to identify the list of productions used during mapping and then attaching the list to each individual.
